# Not all major pathological responses are equal: prognostic heterogeneity and risk stratification after neoadjuvant chemoimmunotherapy in resectable NSCLC

**DOI:** 10.3389/fimmu.2026.1925301

**Published:** 2026-09-25

**Authors:** Jiong Lin, Haijie Xu, Jiming Tang, Chaoquan He, Peishen Li, Xirui Lin, Jiaying Wu, Zexin Huang, Chenshu Huang, Zefeng Xie, Hansheng Wu

**Affiliations:** 1 Department of Thoracic Surgery, The First Affiliated Hospital of Shantou University Medical College, Shantou, China; 2 Shantou University Medical College, Shantou, China; 3 Department of Thoracic Surgery, Guangdong Provincial People’s Hospital (Guangdong Academy of Medical Sciences), Southern Medical University, Guangzhou, China

**Keywords:** lymph node dissection, major pathological response (MPR), neoadjuvant chemoimmunotherapy (NCIT), Non-small cell lung cancer, risk stratification

## Abstract

**Background:**

Major pathological response (MPR) is a widely used efficacy endpoint for neoadjuvant chemoimmunotherapy (NCIT) in resectable non-small cell lung cancer (NSCLC). However, whether MPR without pathological complete response (pCR) represents a prognostically homogeneous population remains unclear, and optimal postoperative management strategies require further exploration. This study evaluated the prognostic heterogeneity, risk stratification, and recurrence patterns of patients achieving MPR.

**Methods:**

This dual-center, retrospective cohort study included 588 patients with resectable NSCLC who received NCIT followed by radical surgery. The primary endpoint was recurrence-free survival (RFS). Patients were categorized into mutually exclusive pCR (n=200), non-pCR MPR (≤10% residual viable tumor without pCR; n = 111), and Non-MPR (n=277) groups. Kaplan-Meier curves and multivariable Cox proportional hazards regression were used to evaluate prognostic factors and develop a risk-stratification system within the MPR subgroup. Additionally, the effect of postoperative adjuvant therapy was evaluated via exploratory subgroup analyses, and anatomical recurrence patterns were systematically compared across response groups.

**Results:**

Recurrence-free survival (RFS) differed among the three groups, with MPR representing an intermediate-risk state. Within the MPR subgroup, baseline cT3–4 stage, postoperative ypN positivity, and lower harvested lymph node count were independently associated with RFS. A simplified system effectively divided MPR patients into low-risk (RFS comparable to pCR) and high-risk groups (RFS approaching Non-MPR). Adjuvant therapy showed no statistically significant RFS benefit in pathological response subgroups. Furthermore, recurrence patterns did not significantly differ among response groups, with brain and bone remaining the predominant distant metastatic sites.

**Conclusions:**

Non-pCR MPR after NCIT is a prognostically heterogeneous state in NSCLC. Stratifying these patients using baseline cT stage, ypN status, and lymph node count refines postoperative risk assessment, highlighting the continued necessity of thorough lymph node dissection. Although adjuvant therapy did not show a definitive RFS benefit in our cohort, its potential value, especially in high-risk MPR patients, warrants prospective validation and should not be interpreted as evidence of lack of benefit. Furthermore, the observed recurrence patterns across response groups, with brain and bone being frequently observed metastatic sites, provide preliminary insights into metastatic distribution after NCIT and may serve as a reference for future surveillance studies.

## Graphical Abstract

**Figure:**
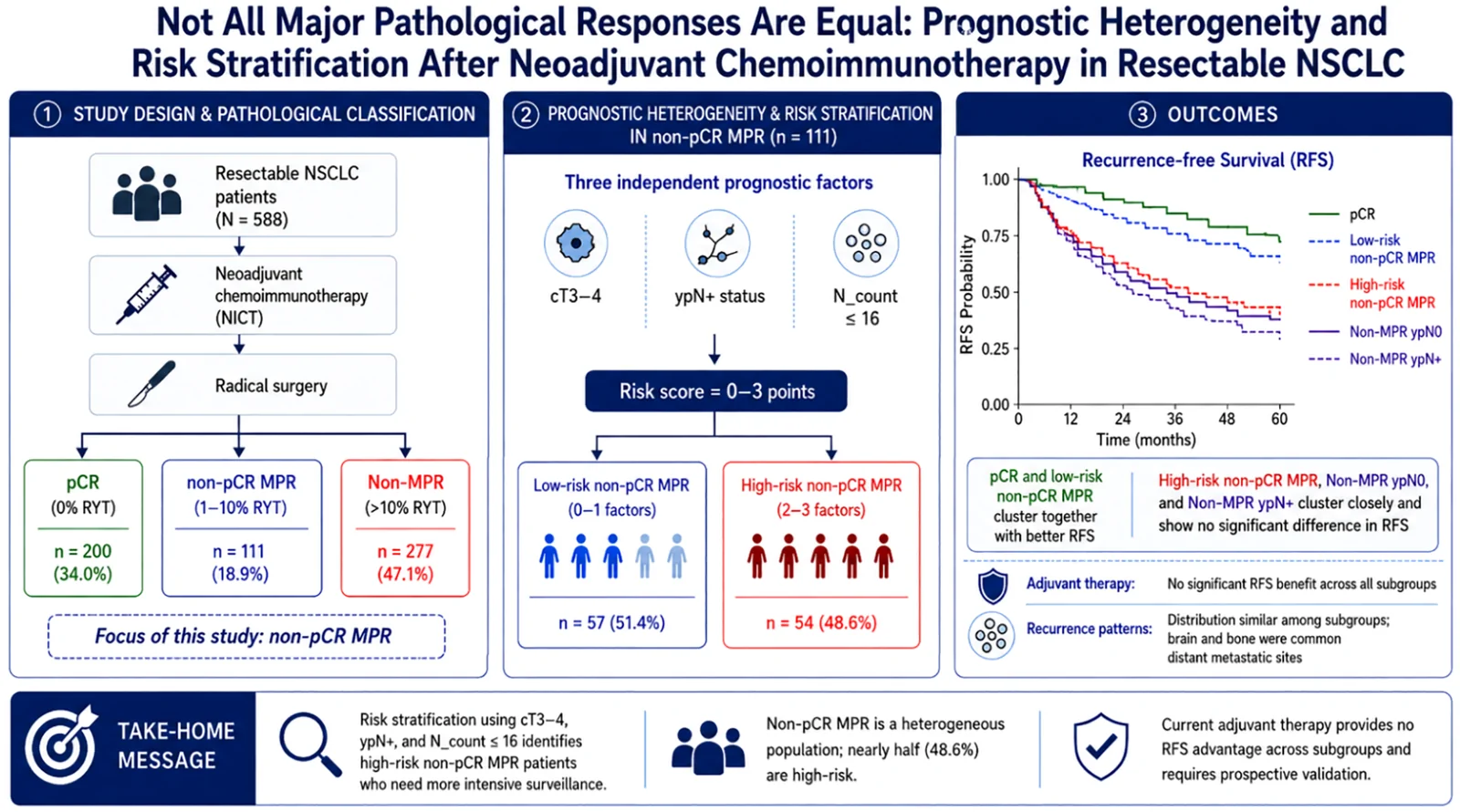

## 1 Introduction

Lung cancer remains one of the most commonly diagnosed malignancies and the leading cause of cancer-related mortality worldwide, representing a major global health burden. Non-small cell lung cancer (NSCLC) accounts for approximately 85% of all lung cancer cases (1). Although radical surgical resection remains the cornerstone of curative-intent treatment for resectable NSCLC (2), many patients present with locally advanced disease at initial diagnosis, for whom upfront surgery alone may no longer be optimal and multimodality treatment is often required (3).

In recent years, neoadjuvant immunotherapy combined with chemotherapy (NCIT), integrating immune checkpoint inhibitors (ICIs) with platinum-based chemotherapy, has become an important treatment strategy for resectable NSCLC (4). Multiple landmark randomized controlled trials (RCTs) have demonstrated that NCIT improves pathological response and surgical feasibility compared with traditional neoadjuvant chemotherapy, leading to improvements in event-free survival (EFS) and overall survival (OS) (5–8). Notably, the updated overall survival analysis of CheckMate 816, which included patients with resectable stage IB–IIIA NSCLC, confirmed a significant OS benefit with neoadjuvant chemoimmunotherapy, providing strong evidence for the use of NCIT across both earlier-stage and locally advanced resectable disease (9).

Pathological response has become an important endpoint for evaluating treatment efficacy after neoadjuvant therapy. Previous studies have shown that pathological complete response (pCR) and major pathological response (MPR) after neoadjuvant chemoimmunotherapy are significantly associated with improved survival outcomes in patients with NSCLC (10). Although long-term survival data from neoadjuvant chemoimmunotherapy trials are still accumulating, evidence regarding its prognostic value remains relatively limited. In this context, pCR and MPR have been widely investigated as indicators of treatment efficacy and potential surrogate endpoints for EFS and OS (11, 12). In particular, the CheckMate 816 study demonstrated a strong association between pCR and long-term survival, further supporting the prognostic value of pathological response after NCIT (9).

However, the proportion of patients achieving pCR remains relatively limited in clinical practice. In contrast, MPR, commonly defined as ≤10% residual viable tumor in the primary lesion, captures a broader population of treatment responders and has therefore been widely used as a pathological endpoint after neoadjuvant therapy (13). Nevertheless, whether MPR represents a prognostically homogeneous low-risk state remains uncertain, as it does not fully account for residual nodal disease, pretreatment tumor burden, the adequacy of lymph node evaluation, or other postoperative risk factors. Moreover, many previous studies have analyzed MPR as a broad responder category that includes pCR, which may obscure the prognostic characteristics of patients who achieve MPR but not pCR (14, 15). Therefore, further risk stratification within the non-pCR MPR population is necessary for more accurate postoperative recurrence-risk assessment.

In addition, the optimal postoperative management of patients achieving favorable pathological response after NCIT remains unclear. Whether postoperative adjuvant therapy provides additional benefit for patients with pCR or MPR is still controversial (16, 17). Meanwhile, studies systematically comparing recurrence patterns among pCR, MPR, and Non-MPR patients remain limited. Whether pathological response mainly affects the probability of recurrence or also influences the anatomical pattern of recurrence requires further investigation.

To address these issues, this dual-center retrospective cohort study evaluated the prognostic value and internal heterogeneity of MPR after NCIT in patients with resectable NSCLC. We compared recurrence-free survival among pCR, non-pCR MPR, and Non-MPR patients, identified prognostic factors within the non-pCR MPR subgroup and, based on these findings, constructed a simplified risk-stratification framework for exploratory purposes, while also exploring postoperative adjuvant therapy and recurrence patterns across pathological response groups.

## 2 Materials and methods

### 2.1 Study design and patient selection

This study was a dual-center, retrospective, real-world cohort study based on consecutive cases from two clinical research centers. Patients with resectable non-small cell lung cancer (NSCLC) who received neoadjuvant chemoimmunotherapy followed by radical surgical resection between January 2019 and December 2024 were included.

The inclusion criteria were as follows: (1) stage IB–III NSCLC according to the 8th edition of the American Joint Committee on Cancer (AJCC) TNM staging system before treatment, assessed by radiological examinations combined with pathological biopsy results; (2) receipt of neoadjuvant therapy consisting of PD-1/PD-L1 inhibitors combined with platinum-based doublet chemotherapy; (3) subsequent radical surgical resection after neoadjuvant therapy; (4) availability of complete baseline and post-neoadjuvant radiological response assessments; and (5) availability of complete postoperative pathological assessment and valid follow-up information.

The exclusion criteria were as follows: (1) non-R0 resection confirmed by postoperative pathology; (2) absence of systematic mediastinal lymph node dissection or sampling; (3) presence of other active malignancies within 5 years; (4) perioperative death due to non-cancer-related causes; and (5) missing key clinical, pathological, or follow-up data that precluded analysis of the primary endpoint.

This study was conducted in accordance with the Declaration of Helsinki and was approved by the Ethics Committees of Guangdong Provincial People’s Hospital (KY2023-351-02) and the First Affiliated Hospital of Shantou University Medical College (B-2025-180). Given the retrospective nature of the study, the requirement for informed consent was waived.

### 2.2 Neoadjuvant treatment, surgery, and radiological assessment

All patients received neoadjuvant chemoimmunotherapy. Immunotherapy consisted of PD-1 or PD-L1 inhibitors, and chemotherapy consisted of platinum-based doublet regimens, including platinum combined with paclitaxel, pemetrexed, gemcitabine, or docetaxel. The specific treatment regimen and number of treatment cycles were determined by the multidisciplinary team according to clinical guidelines, tumor stage, histological subtype, patient performance status, and treatment tolerance.

Surgical evaluation was performed 4–6 weeks after the final cycle of neoadjuvant therapy. Surgical strategies were determined according to preoperative radiological assessment, tumor location, treatment response, and the overall condition of the patient. All patients underwent radical surgical resection with systematic mediastinal lymph node dissection or sampling. Surgical variables included operative time and intraoperative blood loss. Radiological assessment included measurement of the longest diameter of the primary lesion on chest CT before and after neoadjuvant therapy, recorded as CT1_long and CT2_long, respectively. Radiological response was assessed according to the Response Evaluation Criteria in Solid Tumors (RECIST) version 1.1 and dichotomized as complete response or partial response (CR/PR) versus stable disease or progressive disease (SD/PD).

### 2.3 Pathological assessment and data collection

Baseline and treatment-related variables were extracted from electronic medical records, including age, sex, smoking history, body mass index (BMI), number of neoadjuvant treatment cycles, postoperative adjuvant therapy status, clinical T stage, clinical N stage, clinical TNM stage (cTNM stage), tumor location, histology, tumor differentiation, PD-L1 expression status, CT-based longest diameter of the primary lesion before and after neoadjuvant therapy (CT1_long and CT2_long), RECIST-based radiological response, operative time, and intraoperative blood loss. Postoperative pathological specimens were independently reviewed by two senior pathologists according to the standardized pathological assessment recommendations of the International Association for the Study of Lung Cancer (IASLC). Discrepancies were resolved by consensus.

In the main analyses of this study, postoperative pathological response was classified into three mutually exclusive categories: pathological complete response (pCR), major pathological response (MPR), and Non-MPR. pCR was defined as no residual viable tumor cells in both the primary lesion and all examined lymph nodes. MPR was defined as ≤10% residual viable tumor in the primary lesion without achieving pCR. Non-MPR was defined as >10% residual viable tumor in the primary lesion. Other postoperative pathological parameters included post-neoadjuvant pathological nodal status (ypN status), ypTNM stage, number of positive lymph nodes, and total number of harvested lymph nodes (N_count).

### 2.4 Follow-up and study endpoints

The primary endpoint was recurrence-free survival (RFS), defined as the interval from the date of surgery to the first occurrence of disease recurrence or death from any cause. Patients without recurrence or death were censored at the date of last valid follow-up. Postoperative follow-up generally included chest CT, tumor marker assessment, and other imaging examinations when clinically indicated. Follow-up was scheduled every 3–6 months during the first 2 years after surgery, every 6 months during years 3–5, and annually thereafter. Based on clinical symptoms, laboratory findings, or routine imaging results, brain MRI, bone scan, or other systemic imaging examinations were performed when necessary to evaluate distant metastasis.

Recurrence patterns were classified according to the site of first recurrence. Intrathoracic recurrence was defined as recurrence involving the ipsilateral lung, bronchial stump, ipsilateral hilar or mediastinal lymph nodes, pleura, chest wall, or pericardium. Extrathoracic metastasis was defined as metastasis involving the brain, bone, liver, adrenal gland, contralateral lung, supraclavicular or abdominal lymph nodes, or other extrathoracic organs. Recurrence patterns were categorized as intrathoracic recurrence only, extrathoracic metastasis only, or simultaneous intrathoracic recurrence and extrathoracic metastasis.

### 2.5 Statistical analysis

All statistical analyses were performed using R software, version 4.3.2. All tests were two-sided, and a P value < 0.05 was considered statistically significant. Categorical variables were presented as frequencies and percentages and compared using the chi-square test or Fisher’s exact test. Continuous variables were first assessed for normality using the Shapiro-Wilk test. Normally distributed variables were expressed as mean ± standard deviation and compared using the independent-samples t test, whereas non-normally distributed variables were expressed as median and interquartile range (IQR) and compared using the Mann-Whitney U test. Baseline clinicopathological characteristics were compared between the MPR and Non-MPR groups and between the MPR and pCR groups.

Survival curves were generated using the Kaplan-Meier method, and between-group differences in RFS were compared using the log-rank test. Median follow-up time was estimated using the reverse Kaplan–Meier method. Pairwise comparisons of survival curves were adjusted using the Benjamini-Hochberg false discovery rate (FDR) method when more than two groups were compared. RFS was first compared among patients with pCR, MPR, and Non-MPR in the entire cohort. Cox proportional hazards regression models were further used to evaluate the associations between different variables and RFS in the entire cohort. Results were reported as hazard ratios (HRs) with 95% confidence intervals (CIs).

To evaluate prognostic heterogeneity within patients achieving MPR, univariable and multivariable Cox proportional hazards regression analyses were performed in the non-pCR MPR subgroup to identify independent prognostic factors associated with RFS. Variables with P < 0.10 in univariable analysis were included in the multivariable Cox model. The proportional hazards assumption was assessed using Schoenfeld residuals, and multicollinearity was evaluated using the variance inflation factor (VIF), with VIF < 5 considered to indicate no significant multicollinearity.

In the MPR subgroup, a simplified risk-stratification system was constructed based on independent prognostic factors identified by multivariable Cox regression analysis. For continuous variables included in the risk-stratification system, the optimal cutoff value was determined using the surv_cutpoint function in the R package survminer. Each independent prognostic factor was assigned a score of 1 if the risk factor was present and 0 otherwise, and the total risk score was calculated by summing these scores. Patients with MPR were then classified into different risk groups according to the total risk score. The discriminatory performance of the risk-stratification system was evaluated using Kaplan-Meier curves, bootstrap-corrected C-index, and time-dependent area under the curve (AUC). To further assess the clinical relevance of the MPR risk-stratification system, RFS was compared among low-risk MPR, high-risk MPR, pCR, Non-MPR ypN0, and Non-MPR ypN+ groups. Differences in RFS between groups were analyzed using the log-rank test.

The effect of postoperative adjuvant therapy was evaluated using exploratory subgroup analyses. According to pathological response and risk-stratification status, patients were divided into the following subgroups: pCR, low-risk MPR, high-risk MPR, Non-MPR ypN0, and Non-MPR ypN+. Cox regression models were constructed separately within each subgroup to calculate HRs and 95% CIs for postoperative adjuvant therapy versus no adjuvant therapy. Heterogeneity in the effect of adjuvant therapy across subgroups was further assessed by comparing Cox models with and without the subgroup × adjuvant therapy interaction term using the likelihood-ratio test. The association between postoperative adjuvant treatment strategies and recurrence-free survival was further explored using Kaplan–Meier analysis. Patients were categorized according to postoperative treatment strategy, including no adjuvant therapy, adjuvant immunotherapy alone, and adjuvant chemoimmunotherapy followed by maintenance immunotherapy. These analyses were considered exploratory, given the retrospective nature of treatment allocation.

Recurrence pattern analysis included only patients who developed recurrence. According to the site of first recurrence, recurrence patterns were classified as intrathoracic recurrence only, extrathoracic metastasis only, or simultaneous intrathoracic recurrence and extrathoracic metastasis, and the distributions were compared among different pathological response groups. A Sankey diagram was used to visualize the flow from radiological response and pathological response status to first recurrence pattern and was generated using the R package networkD3. Among patients with extrathoracic metastasis, the distribution of specific metastatic sites was further summarized. Center-specific subgroup analyses were performed using the same Kaplan-Meier method to assess the consistency of the risk-stratification system across the two participating centers.

### 2.6 Missing data handling

Missing data were handled as follows: missing values for tumor differentiation and PD-L1 expression were categorized as “Unknown”. Continuous clinical variables with a missing proportion <5% were imputed using the median, whereas other variables were analyzed using complete-case analysis. All missing data handling procedures were performed before the corresponding statistical analyses.

## 3 Results

### 3.1 Baseline characteristics and pathological response distribution

A total of 588 patients with resectable non-small cell lung cancer (NSCLC) who received neoadjuvant chemoimmunotherapy (NCIT) followed by radical surgical resection were included in this study. The patient selection process is shown in **Figure 1**. The median age of the overall cohort was 62.0 years, 480 patients (81.6%) were male, and 303 patients (51.5%) had a history of smoking. Before treatment, 436 patients (74.1%) had clinical stage III disease. Regarding histological subtype, lung squamous cell carcinoma (LUSC) and lung adenocarcinoma (LUAD) accounted for 270 (45.9%) and 249 (42.3%) patients, respectively. According to postoperative pathological response, patients were classified into three mutually exclusive categories: 200 patients (34.0%) achieved pathological complete response (pCR), 111 patients (18.9%) achieved major pathological response without pCR (MPR), and 277 patients (47.1%) did not achieve MPR (Non-MPR). The baseline clinical, radiological, surgical, and pathological characteristics of the overall cohort and the three pathological response groups are summarized in **Table 1**.

**Figure 1:**
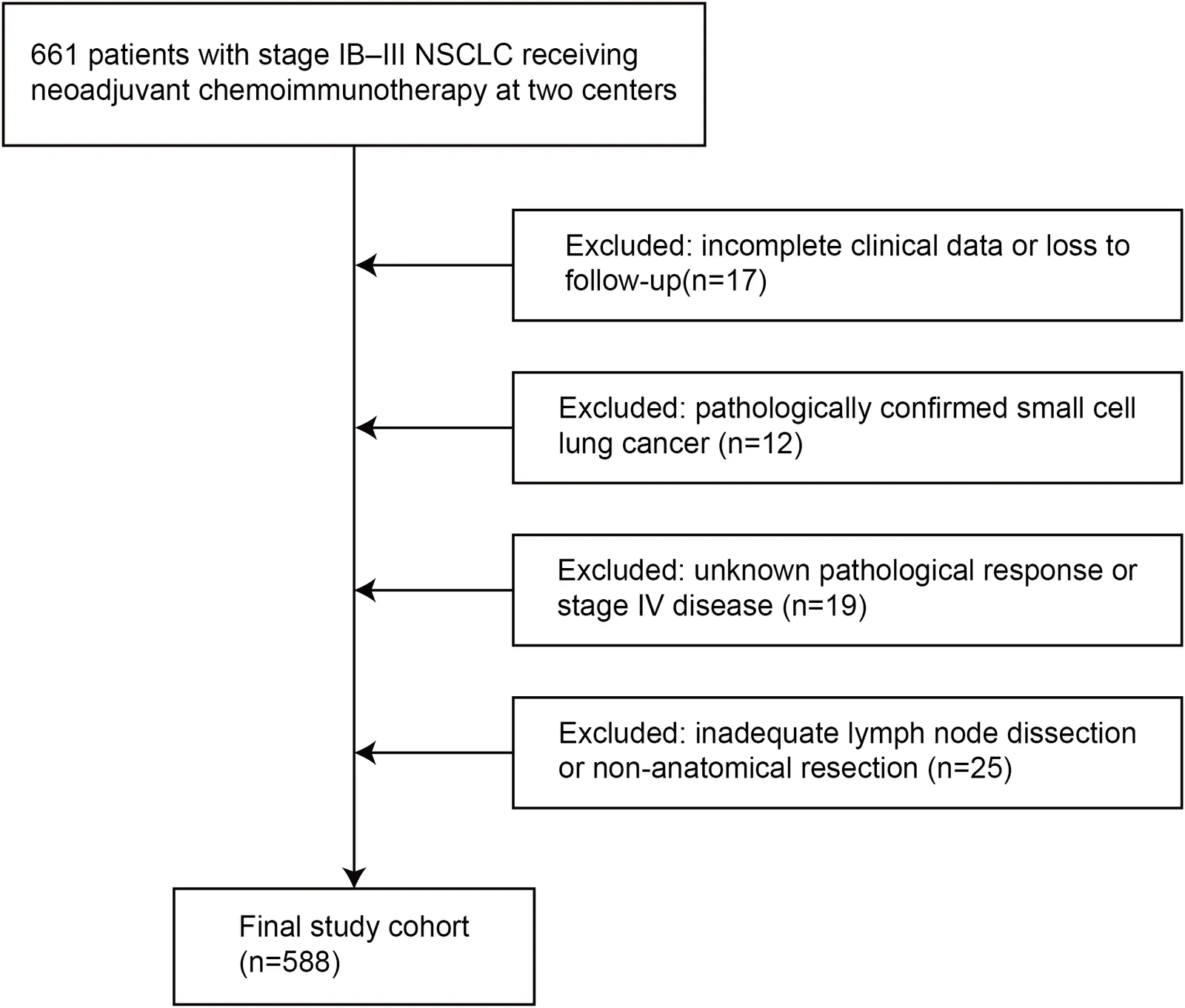
Patient selection flowchart. Flowchart showing the screening process and final inclusion of patients with resectable non-small cell lung cancer (NSCLC) who received neoadjuvant chemoimmunotherapy (NCIT) followed by radical surgery. Patients were classified into pathological complete response (pCR), major pathological response (MPR), and Non-MPR groups according to postoperative pathological response. In this study, MPR referred to patients with ≤10% residual viable tumor in the primary lesion who did not achieve pCR.

**Table 1:** Baseline clinical, radiological, surgical, and pathological characteristics of the overall cohort and according to pathological response.

Characteristics	Overall (n=588)	MPR (n=111)	Non-MPR (n=277)	pCR (n=200)	P value^a^	P value^b^
Sex					0.765	0.030
Female	108 (18.4)	23 (20.7)	63 (22.7)	22 (11.0)		
Male	480 (81.6)	88 (79.3)	214 (77.3)	178 (89.0)		
Age					1.000	0.611
>65	197 (33.5)	36 (32.4)	89 (32.1)	72 (36.0)		
≤65	391 (66.5)	75 (67.6)	188 (67.9)	128 (64.0)		
BMI	23.28 [21.09, 25.03]	22.66 [20.85, 24.61]	23.38 [21.09, 25.40]	23.15 [21.22, 24.98]	0.193	0.386
Smoking					0.043	0.315
Non-smoker	285 (48.5)	50 (45.0)	158 (57.0)	77 (38.5)		
Smoker	303 (51.5)	61 (55.0)	119 (43.0)	123 (61.5)		
Neoadjuvant cycles					0.659	0.932
<3	120 (20.4)	20 (18.0)	61 (22.0)	39 (19.5)		
3-4	426 (72.4)	83 (74.8)	195 (70.4)	148 (74.0)		
>4	42 (7.1)	8 (7.2)	21 (7.6)	13 (6.5)		
Adjuvant therapy					0.085	1.000
No	190 (32.3)	41 (36.9)	76 (27.4)	73 (36.5)		
Yes	398 (67.7)	70 (63.1)	201 (72.6)	127 (63.5)		
RECIST					<0.001	0.228
PD_SD	231 (39.3)	32 (28.8)	155 (56.0)	44 (22.0)		
CR_PR	357 (60.7)	79 (71.2)	122 (44.0)	156 (78.0)		
cT stage					0.274	1.000
T1-2	300 (51.0)	53 (47.7)	151 (54.5)	96 (48.0)		
T3-4	288 (49.0)	58 (52.3)	126 (45.5)	104 (52.0)		
cN stage					0.584	0.447
N0	101 (17.2)	16 (14.4)	48 (17.3)	37 (18.5)		
N+	487 (82.8)	95 (85.6)	229 (82.7)	163 (81.5)		
cTNM stage					0.023	0.022
I-II	152 (25.9)	18 (16.2)	77 (27.8)	57 (28.5)		
III	436 (74.1)	93 (83.8)	200 (72.2)	143 (71.5)		
Differentiation					<0.001	<0.001
G1-2	204 (35.0)	48 (43.2)	129 (46.6)	29 (14.5)		
G3	204 (34.7)	36 (32.4)	130 (46.9)	38 (19.0)		
Gx	178 (30.3)	27 (24.3)	18 (6.5)	133 (66.5)		
Histology					0.036	0.128
LUAD	249 (42.3)	44 (39.6)	148 (53.4)	57 (28.5)		
LUSC	270 (45.9)	55 (49.5)	100 (36.1)	115 (57.5)		
Others	69 (11.7)	12 (10.8)	29 (10.5)	28 (14.0)		
ypT					<0.001	<0.001
0	214 (36.4)	14 (12.6)	0 (0.0)	200 (100.0)		
1	274 (46.6)	97 (87.4)	177 (63.9)	0 (0.0)		
2	62 (10.5)	0 (0.0)	62 (22.4)	0 (0.0)		
3	25 (4.3)	0 (0.0)	25 (9.0)	0 (0.0)		
4	13 (2.2)	0 (0.0)	13 (4.7)	0 (0.0)		
ypN					0.136	<0.001
N0	445 (75.7)	77 (69.4)	168 (60.6)	200 (100.0)		
N+	143 (24.3)	34(30.6)	109 (39.4)	0 (0.0)		
ypTNM stage					0.001	<0.001
0	200 (34.0)	0 (0.0)	0 (0.0)	200 (100.0)		
I	211 (35.9)	77 (69.4)	134 (48.4)	0 (0.0)		
II	109 (18.5)	22 (19.8)	87 (31.4)	0 (0.0)		
III	68 (11.6)	12 (10.8)	56 (20.2)	0 (0.0)		
Tumor site					0.398	0.301
Central	227 (38.6)	43 (38.7)	93 (33.6)	91 (45.5)		
Peripheral	361 (61.4)	68 (61.3)	184 (66.4)	109 (54.5)		
PD-L1					0.102	0.066
Strong positive	70 (11.9)	18 (16.2)	24 (8.7)	28 (14.0)		
Positive	154 (26.2)	31 (27.9)	89 (32.1)	34 (17.0)		
Negative	128 (21.8)	21 (18.9)	70 (25.3)	37 (18.5)		
Unknown	236 (40.1)	41 (36.9)	94 (33.9)	101 (50.5)		
N_count	14.00 [10.00, 19.00]	14.00 [9.50, 19.00]	15.00 [10.00, 19.00]	14.00 [10.00, 19.00]	0.738	0.556
CT1_long	4.80 [3.48, 7.10]	5.20 [3.70, 7.05]	4.60 [3.20, 7.10]	4.90 [3.58, 7.10]	0.340	0.765
CT2_long	2.60 [1.60, 3.60]	2.70 [1.80, 3.30]	2.80 [1.80, 3.80]	2.30 [1.30, 3.20]	0.257	0.039
Operative time	196.00 [150.00, 250.00]	185.00 [150.00, 235.50]	197.00[155.00, 253.00]	198.00 [150.75, 250.00]	0.217	0.389
Blood loss	50.00 [20.00, 100.00]	30.00 [20.00, 50.00]	50.00 [20.00, 100.00]	30.00 [20.00, 100.00]	0.057	0.223

Data are presented as n (%) or median [interquartile range], as appropriate. In this study, pCR, MPR, and Non-MPR were defined as mutually exclusive pathological response categories; MPR referred to patients with ≤10% residual viable tumor in the primary lesion who did not achieve pCR.

^a P value for the comparison between the MPR and Non-MPR groups.

^b P value for the comparison between the MPR and pCR groups.

BMI, body mass index; cN, clinical nodal stage; cT, clinical tumor stage; CR, complete response; IQR, interquartile range; LUAD, lung adenocarcinoma; LUSC, lung squamous cell carcinoma; MPR, major pathological response; N_count, total number of harvested lymph nodes; Non-MPR, non-major pathological response; pCR, pathological complete response; PD, progressive disease; PD-L1, programmed death-ligand 1; PR, partial response; RECIST, Response Evaluation Criteria in Solid Tumors; SD, stable disease; ypN, post-neoadjuvant pathological nodal status; ypTNM, post-neoadjuvant pathological TNM stage.

In pairwise comparisons using the MPR group as the reference group, no significant differences were observed between the MPR group and either the Non-MPR or pCR groups in age, BMI, number of neoadjuvant treatment cycles, cT stage, cN stage, tumor location, PD-L1 expression status, total number of harvested lymph nodes (N_count), baseline longest diameter of the primary lesion on CT (CT1_long), operative time, or intraoperative blood loss. Compared with the Non-MPR group, the MPR group had a higher proportion of smokers (55.0% vs. 43.0%, P = 0.043), a higher proportion of LUSC (49.5% vs. 36.1%, P = 0.036), and a higher proportion of clinical stage III disease (83.8% vs. 72.2%, P = 0.023). Compared with the pCR group, the MPR group had a higher proportion of female patients (20.7% vs. 11.0%, P = 0.030) and a higher proportion of clinical stage III disease (83.8% vs. 71.5%, P = 0.022).

Regarding radiological response, the proportion of patients with CR/PR according to RECIST was significantly higher in the MPR group than in the Non-MPR group (71.2% vs. 44.0%, P < 0.001), but did not differ significantly between the MPR and pCR groups (71.2% vs. 78.0%, P = 0.228). The post-neoadjuvant longest diameter of the primary lesion on CT (CT2_long) did not differ significantly between the MPR and Non-MPR groups (P = 0.257), but was higher in the MPR group than in the pCR group (median, 2.70 cm vs. 2.30 cm; P = 0.039). Regarding postoperative pathological characteristics, the ypN-positive rate was 30.6% in the MPR group and 39.4% in the Non-MPR group, with no significant difference between the two groups (P = 0.136). Tumor differentiation differed significantly between the MPR group and both the Non-MPR and pCR groups (both P < 0.001), although the proportion of unknown differentiation was relatively high in the pCR group. Postoperative adjuvant therapy, operative time, and intraoperative blood loss did not differ significantly between the MPR group and the other two groups.

### 3.2 Recurrence-free survival according to pathological response

During a median follow-up of 20.5 months (95% CI, 19.1–21.5 months), 164 recurrence events were recorded in the overall cohort. Kaplan-Meier curves showed significant differences in RFS among the pCR, MPR, and Non-MPR groups (overall P < 0.001; **Figure 2**). The pCR group had the most favorable prognosis, the Non-MPR group had the poorest prognosis, and the MPR group showed an intermediate-risk pattern. Pairwise comparisons showed that RFS was significantly better in the pCR group than in the MPR group (P = 0.009), and significantly better in the MPR group than in the Non-MPR group (P = 0.016). In Cox regression analyses of the overall cohort, pathological response status was associated with RFS (**Supplementary Table S1**). Compared with the pCR group, the Non-MPR group had a significantly increased risk of recurrence (HR = 2.35, 95% CI: 1.47–3.76, P < 0.001), whereas the MPR group showed a trend toward increased recurrence risk that did not reach conventional statistical significance (HR = 1.68, 95% CI: 0.99–2.85, P = 0.056). In addition, postoperative ypN positivity was associated with a higher recurrence risk (HR = 1.50, 95% CI: 1.05–2.13, P = 0.025), whereas a higher total number of harvested lymph nodes was associated with a lower recurrence risk (per 1-node increase, HR = 0.96, 95% CI: 0.94–0.98, P < 0.001).

**Figure 2:**
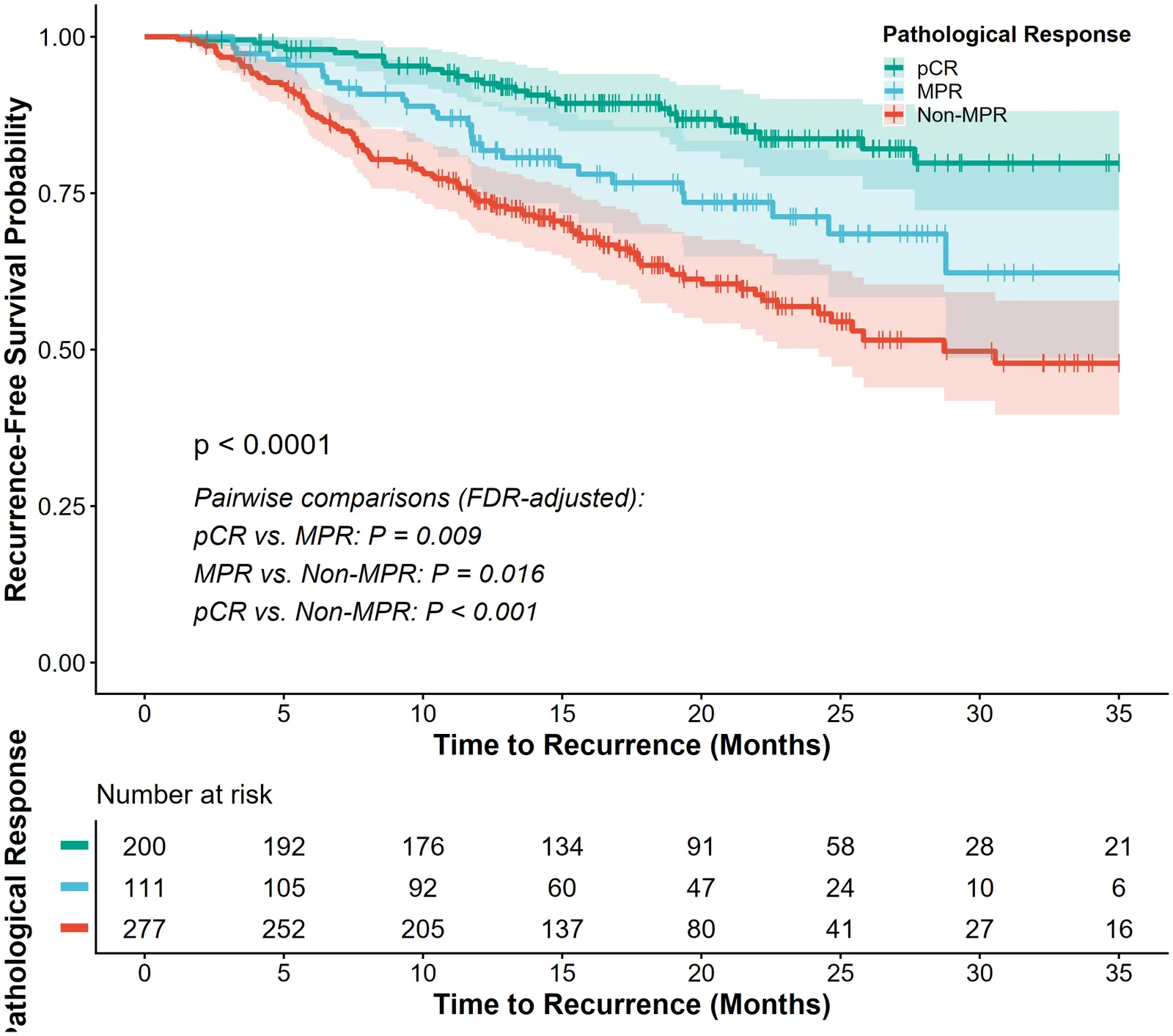
Recurrence-free survival according to pathological response. Kaplan–Meier curves comparing recurrence-free survival (RFS) among patients with pCR, MPR, and Non-MPR. Significant differences in RFS were observed among the three pathological response groups, with pCR showing the most favorable prognosis, Non-MPR showing the poorest prognosis, and MPR showing an intermediate-risk pattern. Pairwise log-rank P values are shown in the image.

### 3.3 Prognostic heterogeneity within the MPR subgroup

Among the 111 patients who achieved MPR but not pCR, univariable Cox regression analysis showed that baseline cT stage, postoperative ypN status, and total number of harvested lymph nodes (N_count) were associated with RFS (all P < 0.10). Multivariable Cox regression analysis further showed that baseline cT3–4 (HR = 2.57, 95% CI: 1.12–5.90, P = 0.026), postoperative ypN positivity (HR = 3.16, 95% CI: 1.47–6.80, P = 0.003), and N_count (per 1-node increase, HR = 0.93, 95% CI: 0.87–0.99, P = 0.024) were independent factors associated with RFS in the MPR subgroup (**Table 2**). Kaplan-Meier curves showed that patients with postoperative ypN positivity had significantly worse RFS than those with ypN0 disease (log-rank P = 0.041; **Figure 3A**). Using the surv_cutpoint function, the optimal cutoff value for N_count was identified as 16, and patients with N_count ≤16 had significantly worse RFS than those with N_count >16 (log-rank P = 0.028; **Figure 3B**). In addition, patients with baseline cT3–4 disease had significantly worse RFS than those with cT1–2 disease (log-rank P = 0.031; **Figure 3C**).

**Table 2:** Univariate and multivariate cox regression for RFS in non-pcr MPR subgroup.

		Univariate		Multivariate	
Variables	Levels	HR (95% CI)	P value	HR (95% CI)	P value
Sex	Male	Reference	0.809		
	Female	0.89 (0.34-2.34)	0.809		
Age	≤65	Reference	0.838		
	>65	1.08 (0.50-2.33)	0.838		
Smoking	Non-smoker	Reference	0.707		
	Smoker	1.15 (0.55-2.42)	0.707		
BMI	Per 1 unit increase	0.99 (0.87-1.13)	0.892		
Adjuvant therapy	No	Reference	0.748		
	Yes	0.88 (0.40-1.92)	0.748		
Neoadjuvant cycles	<3	Reference	0.419		
	3-4	0.72 (0.30-1.72)	0.459		
	>4	1.53 (0.39-6.00)	0.545		
Histology	LUAD	Reference	0.332		
	LUSC	0.78 (0.37-1.64)	0.515		
	Others	0.23 (0.03-1.73)	0.153		
Differentiation	G1-2	Reference	0.576		
	G3	0.96 (0.39-2.37)	0.933		
	Unknown	1.51 (0.63-3.62)	0.361		
Tumor site	Peripheral	Reference	0.943		
	central	0.97 (0.46-2.07)	0.943		
cT stage	T1-2	Reference	0.033	Reference	
	T3-4	2.43 (1.07-5.51)	0.033	2.57 (1.12-5.90)	0.026
cN stage	N0	Reference	0.903		
	N+	0.94 (0.32-2.70)	0.903		
ypN stage	N0	Reference	0.022	Reference	
	N+	2.35 (1.13-4.88)	0.022	3.16 (1.47-6.80)	0.003
ypT stage	T0	Reference	0.300		
	T1	0.60 (0.23-1.58)	0.300		
cTNM stage	I-II	Reference	0.320		
	III	0.63 (0.26-1.56)	0.320		
CT1_long	Per 1 unit increase	0.97 (0.84-1.13)	0.721		
CT2_long	Per 1 unit increase	1.12 (0.85-1.47)	0.434		
RECIST	PD_SD	Reference	0.535		
	CR_PR	1.30 (0.57-2.97)	0.535		
PD-L1	Negative	Reference	0.191		
	Strong positive	1.89 (0.60-5.96)	0.279		
	Positive	0.53 (0.15-1.83)	0.314		
	Unknown	0.92 (0.31-2.71)	0.885		
Operative time	Per 1 unit increase	1.00 (0.99-1.00)	0.712		
Blood loss	Per 1 unit increase	1.00 (1.00-1.00)	0.699		
N_count	Per 1 unit increase	0.94 (0.89-1.00)	0.046	0.93 (0.87-0.99)	0.024

MPR subgroup included patients with ≤10% residual viable tumor in the primary lesion who did not achieve pCR. Hazard ratios (HRs) and 95% confidence intervals (CIs) were estimated using Cox proportional hazards regression models. Variables with P < 0.10 in univariable analysis were considered for multivariable analysis.

CI, confidence interval; HR, hazard ratio; LUAD, lung adenocarcinoma; LUSC, lung squamous cell carcinoma; MPR, major pathological response; N_count, total number of harvested lymph nodes; pCR, pathological complete response; PD-L1, programmed death-ligand 1; RECIST, Response Evaluation Criteria in Solid Tumors; RFS, recurrence-free survival.

**Figure 3:**
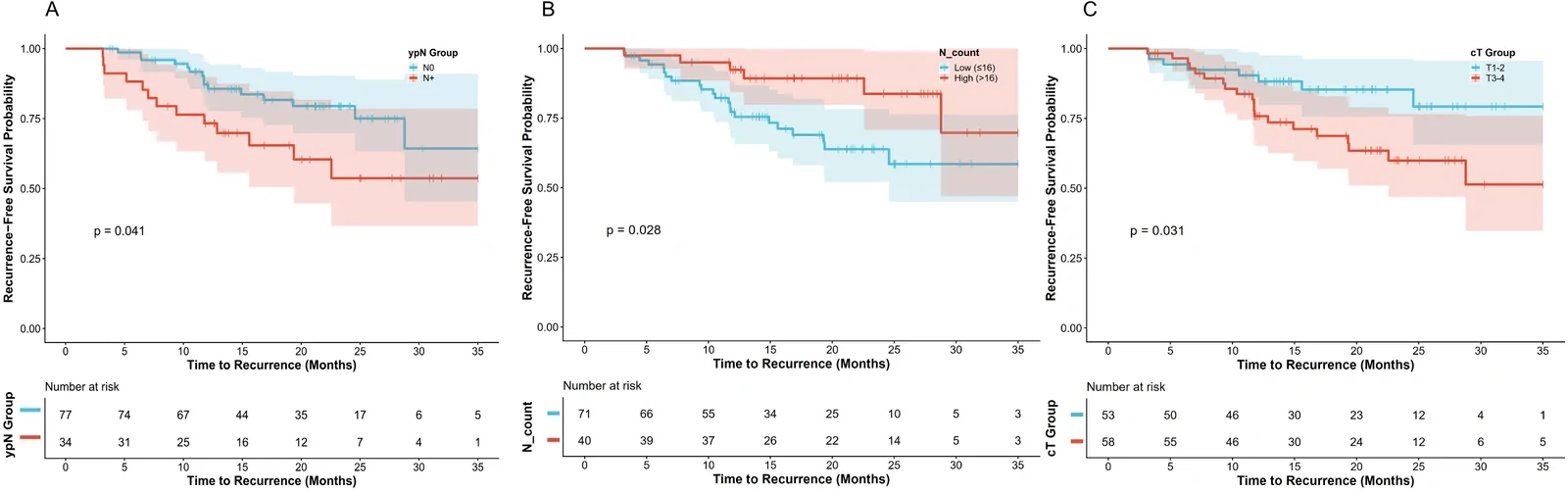
Prognostic factors for recurrence-free survival within the MPR subgroup. Kaplan–Meier curves showing RFS stratified by independent prognostic factors in patients who achieved MPR but not pCR. **(A)** RFS according to postoperative nodal status, comparing ypN0 and ypN+ patients. **(B)** RFS according to the number of harvested lymph nodes, using 16 lymph nodes as the optimal cutoff. **(C)** RFS according to baseline clinical T stage, comparing cT1–2 and cT3–4. Log-rank P values are shown in each panel.

### 3.4 Development and evaluation of the MPR risk-stratification system

A simplified risk-stratification system was constructed based on the three independent prognostic factors identified in the multivariable Cox regression analysis of the MPR subgroup. Baseline cT3–4, postoperative ypN positivity, and N_count ≤16 were each assigned 1 point. According to the number of risk factors, patients with MPR were divided into low-risk and high-risk groups. The low-risk group was defined as having 0–1 risk factor, and the high-risk group was defined as having 2–3 risk factors. **Figure 4A** shows the distribution and intersections of the three risk factors in the MPR subgroup. **Figure 4B** and **Supplementary Table S2** showed that RFS did not differ significantly between the low-risk MPR and pCR groups (P = 0.856). The high-risk MPR group had significantly worse RFS than the low-risk MPR group (P = 0.009) and the pCR group (P < 0.001). No significant differences in RFS were observed between the high-risk MPR group and the Non-MPR ypN0 or Non-MPR ypN+ groups (P = 0.856 and P = 0.504, respectively).

**Figure 4:**
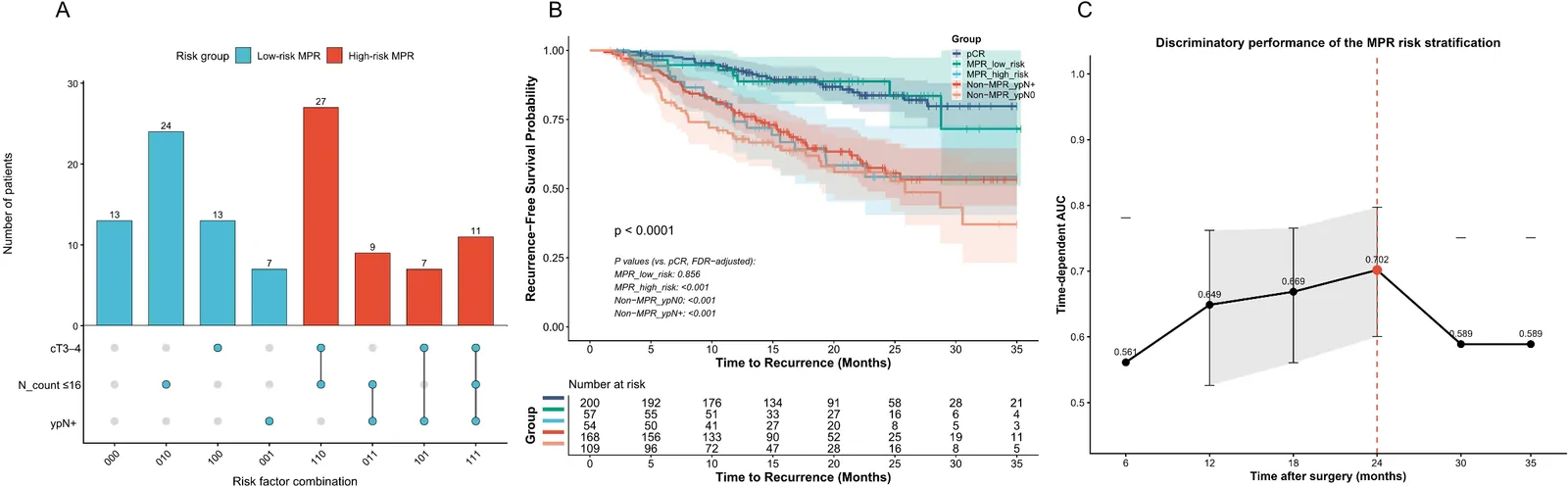
Development and performance of the MPR risk-stratification system. **(A)** UpSet plot showing the distribution and intersections of the three independent prognostic factors within the MPR subgroup: baseline cT3–4 stage, postoperative nodal positivity, and N_count ≤16. **(B)** Kaplan–Meier curves comparing RFS among pCR, low-risk MPR, high-risk MPR, Non-MPR ypN0, and Non-MPR ypN+ groups. Low-risk MPR patients showed RFS comparable to pCR patients, whereas high-risk MPR patients showed RFS close to that of Non-MPR patients. **(C)** Bootstrap internal validation of the MPR risk-stratification system using time-dependent AUC analysis. The curve shows the discriminatory performance of the risk-stratification system for predicting recurrence-free survival at different postoperative time points.

The bootstrap-corrected C-index of the risk-stratification system was 0.637 (95% CI: 0.543–0.727). As shown in **Figure 4C**, the time-dependent AUC reached its highest value at 24 months after surgery, with an AUC of 0.702. Center-specific Kaplan-Meier analyses suggested generally consistent risk stratification trends across the two centers, though not statistically significant in the smaller cohort (**Supplementary Figure S2**). Further subgroup analysis according to histological subtype showed that the risk-stratification system significantly discriminated RFS in patients with LUAD (P = 0.015), while a separation trend was observed in patients with LUSC (P = 0.093; **Supplementary Figure S1**).

### 3.5 Subgroup analysis of postoperative adjuvant therapy

To evaluate the role of postoperative adjuvant therapy across different pathological response and risk-stratified subgroups, Cox regression analyses were performed separately in the pCR, low-risk MPR, high-risk MPR, Non-MPR ypN0, and Non-MPR ypN+ subgroups. As shown in **Figure 5**, in this exploratory subgroup analysis, postoperative adjuvant therapy was not significantly associated with RFS in any subgroup, with all 95% CIs crossing 1. Further analysis using a Cox model including the subgroup × adjuvant therapy interaction term showed no significant interaction between pathological response/risk-stratification status and postoperative adjuvant therapy (P for interaction = 0.510). Given the non-randomized treatment allocation, heterogeneous regimens, and limited subgroup event numbers, these findings should be interpreted cautiously. When further stratified by adjuvant therapy type, neither immunotherapy alone nor chemoimmunotherapy followed by maintenance immunotherapy was significantly associated with RFS in exploratory analyses (**Supplementary Figure S3**).

**Figure 5:**
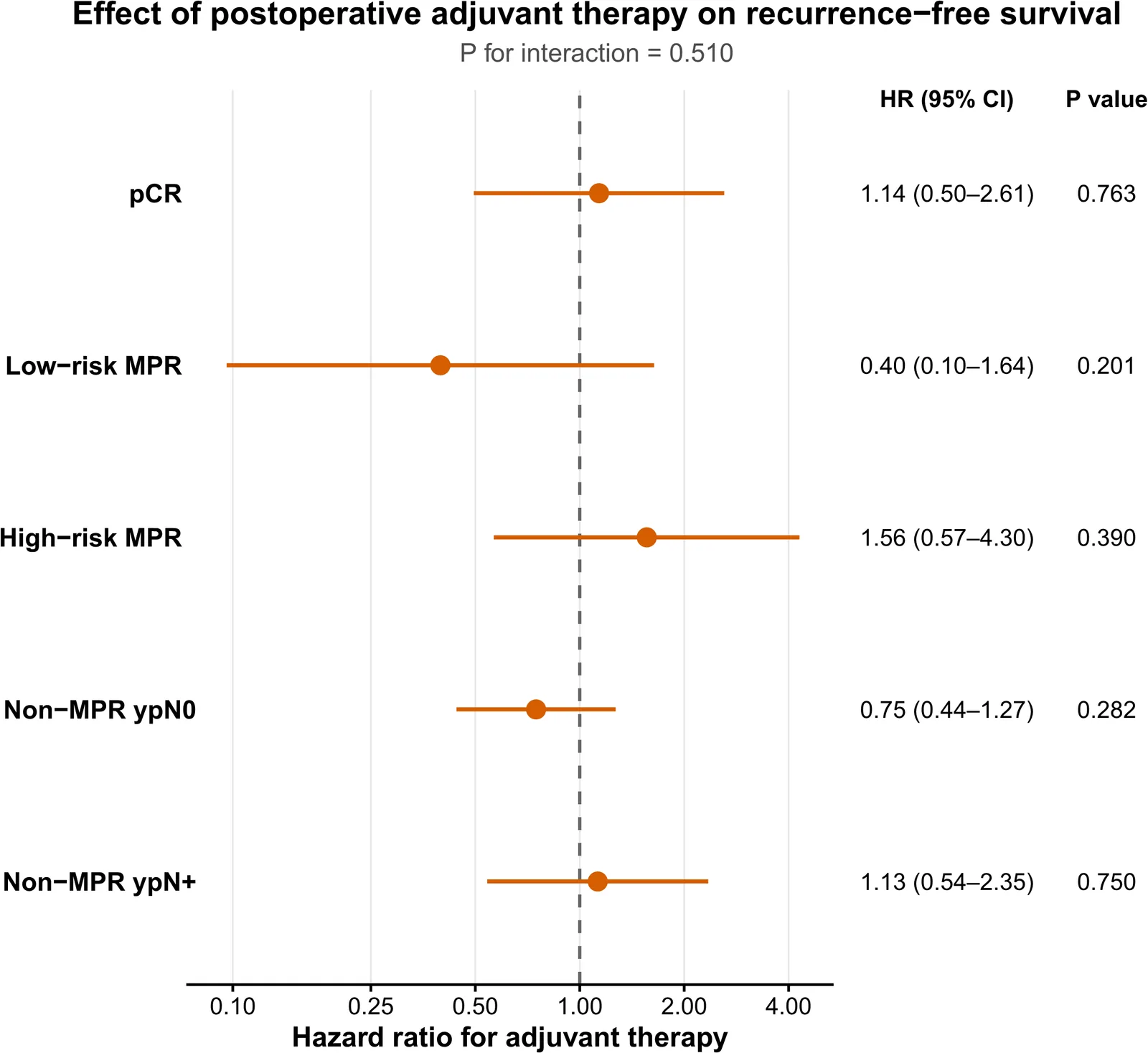
Subgroup analysis of postoperative adjuvant therapy. Forest plot showing the association between postoperative adjuvant therapy and RFS across pathological response and risk-defined subgroups, including pCR, low-risk MPR, high-risk MPR, Non-MPR ypN0, and Non-MPR ypN+. Hazard ratios (HRs) and 95% confidence intervals (CIs) were estimated using Cox proportional hazards models. The vertical reference line indicates HR = 1. No significant RFS benefit of postoperative adjuvant therapy was observed in any subgroup, and no significant subgroup-by-adjuvant therapy interaction was detected.

### 3.6 Recurrence pattern analysis

In the overall cohort, the recurrence rates in the pCR, MPR, and Non-MPR groups were 15.0%, 26.1%, and 37.9%, respectively (**Table 3**). Compared with the MPR group, the pCR group had a significantly lower recurrence rate (P = 0.025), whereas the Non-MPR group had a significantly higher recurrence rate (P = 0.037). Among the 164 patients who developed recurrence, the first recurrence pattern was further analyzed. The proportions of intrathoracic recurrence only in the MPR, pCR, and Non-MPR groups were 41.4%, 43.3%, and 41.0%, respectively. The proportions of extrathoracic metastasis only were 37.9%, 40.0%, and 45.7%, respectively, and the proportions of simultaneous intrathoracic recurrence and extrathoracic metastasis were 20.7%, 16.7%, and 13.3%, respectively. The distribution of first recurrence patterns did not differ significantly among the pathological response groups (MPR vs. pCR, P = 0.924; MPR vs. Non-MPR, P = 0.566; **Table 3**). **Figure 6A** shows the relationship among radiological response, pathological response status, and first recurrence pattern in patients who developed recurrence. Among recurrent patients with extrathoracic metastasis, **Figure 6B** shows the distribution of extrathoracic metastatic sites. Brain and bone were the most common extrathoracic metastatic sites. The proportions of brain metastasis in the MPR, Non-MPR, and pCR groups were 47.1%, 40.3%, and 41.2%, respectively, and the proportions of bone metastasis were 35.3%, 25.8%, and 17.6%, respectively.

**Table 3:** Radiological response, recurrence status, and recurrence patterns according to pathological response.

Variables	MPR patients	pCR patients	P value^a^	Non-MPR patients	P value^b^
Radiological response (CR+PR)			0.228		<0.001
Yes	79 (71.2%)	156 (78.0%)		122 (44.0%)	
No	32 (28.8%)	44 (22.0%)		155 (56.0%)	
Recurrence status			0.025		0.037
Recurrence-free	82 (73.9%)	170 (85.0%)		172 (62.1%)	
Recurrence	29 (26.1%)	30 (15.0%)		105 (37.9%)	
Recurrence pattern			0.924		0.566
Intrathoracic recurrence only	12 (41.4%)	13 (43.3%)		43 (41.0%)	
Extrathoracic metastasis only	11 (37.9%)	12 (40.0%)		48 (45.7%)	
Simultaneous intrathoracic and extrathoracic	6 (20.7%)	5 (16.7%)		14 (13.3%)	

Data are presented as n (%). Recurrence patterns were analyzed among patients who developed recurrence. In this study, MPR referred to patients with ≤10% residual viable tumor in the primary lesion who did not achieve pCR.

^a P value for the comparison between the MPR and pCR groups.

^b P value for the comparison between the MPR and Non-MPR groups.

CR, complete response; MPR, major pathological response; Non-MPR, non-major pathological response; pCR, pathological complete response; PD, progressive disease; PR, partial response; RECIST, Response Evaluation Criteria in Solid Tumors; SD, stable disease.

**Figure 6:**
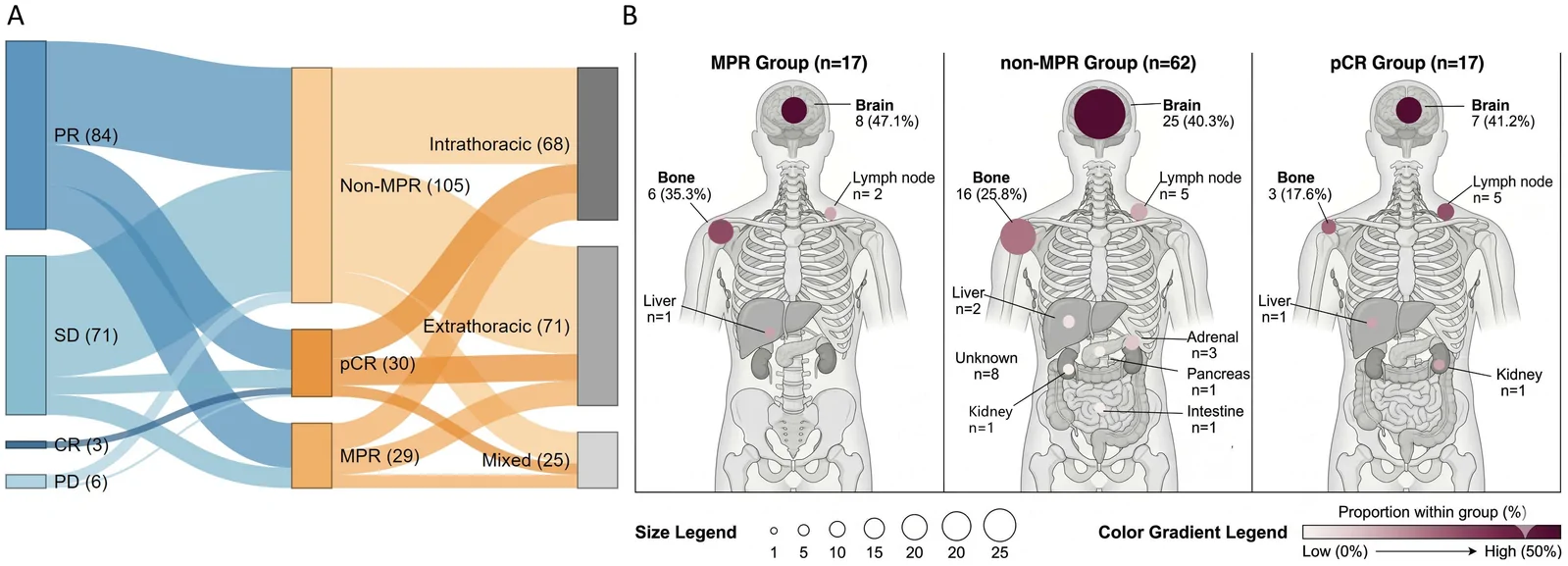
Recurrence patterns and extrathoracic metastatic sites according to pathological response. **(A)** Sankey diagram illustrating the relationship among radiological response, pathological response status, and first recurrence pattern among patients who developed recurrence. Recurrence patterns were classified as intrathoracic recurrence only, extrathoracic metastasis only, or simultaneous intrathoracic recurrence and extrathoracic metastasis. **(B)** Distribution of extrathoracic metastatic sites among recurrent patients with extrathoracic metastasis, stratified by pathological response group. Brain and bone were the most common extrathoracic metastatic sites across groups.

## 4 Discussion

In this dual-center retrospective cohort study of patients with resectable NSCLC who underwent neoadjuvant chemoimmunotherapy followed by radical surgery, we systematically evaluated the prognostic value and internal heterogeneity of MPR. The main findings were as follows. First, pCR, MPR, and Non-MPR patients showed clearly stratified recurrence-free survival (RFS), with MPR patients having an intermediate recurrence risk between pCR and Non-MPR patients. Second, among patients who achieved MPR but not pCR, substantial prognostic heterogeneity remained. cT3–4, residual nodal positivity, and a lower number of harvested lymph nodes were significantly associated with RFS. Third, a simplified risk-stratification system based on these three factors further classified MPR patients into low- and high-risk groups. Low-risk MPR patients had RFS comparable to that of pCR patients, whereas high-risk MPR patients had RFS similar to that of Non-MPR patients. Fourth, postoperative adjuvant therapy was not associated with significant RFS improvement in any pathological response or risk-defined subgroup, and no significant interaction was observed. Finally, although recurrence rates differed across pathological response groups, recurrence patterns among recurrent patients were broadly similar, with brain and bone being common distant metastatic sites.

One of the central findings of this study is that patients achieving MPR are not prognostically homogeneous. Unlike previous studies that analyzed pCR as a subset of MPR, the present study defined pCR, MPR, and Non-MPR as mutually exclusive pathological response categories, thereby allowing a more precise evaluation of the prognostic features of patients who achieved MPR but not pCR. Our results showed that non-pCR MPR patients had significantly better RFS than Non-MPR patients but worse RFS than pCR patients, representing an intermediate-risk state between these two groups. This finding is consistent with the results reported by Nuccio et al. (18). Although combining pCR and MPR may increase the analyzable sample size, this approach may obscure prognostic differences within the non-pCR MPR subgroup and limit accurate recurrence-risk assessment in this population (19, 20). MPR is defined only by the proportion of residual viable tumor in the primary lesion (≤10%) and does not account for residual nodal disease, minimal residual disease (MRD), or other high-risk pathological features. Based on a relatively large MPR cohort, our study further demonstrated that even when the primary lesion reaches MPR, postoperative outcomes remain significantly influenced by multidimensional factors, including baseline cT stage, postoperative ypN status, and the adequacy of lymph node dissection. Therefore, integrating additional clinicopathological factors to further stratify MPR patients is clinically important for more accurate recurrence-risk assessment and individualized management.

In the MPR subgroup, we identified three independent prognostic factors: postoperative nodal positivity (ypN+), lower lymph node count (N_count ≤16), and baseline cT3–4 stage. These findings are consistent with previous studies; importantly, to our knowledge, these factors are among the first to be systematically evaluated specifically in the non-pCR MPR population. Nodal status was a key factor influencing prognosis in MPR patients. Notably, in the clinicopathological comparison, the ypN-positive rate was not significantly different between the MPR and Non-MPR groups. Although this finding should not be interpreted as evidence of equivalence, it suggests that favorable pathological regression in the primary lesion may not fully reflect the risk of residual nodal disease. In our multivariable analysis, postoperative nodal positivity was an independent risk factor for recurrence, with ypN+ patients having a 3.16-fold higher recurrence risk than ypN0 patients. This result is consistent with previous studies. Shao et al. and Xu et al. developed prognostic stratification models incorporating pathological response and ypN status, both showing that postoperative nodal positivity was associated with increased recurrence risk (14, 21). Ma et al. further confirmed in a multicenter study that patients with ypN0 disease had better outcomes than those with ypN+ disease regardless of pretreatment nodal status, and that among patients achieving MPR, those with ypN0 disease had significantly better DFS than those with ypN+ disease (15). However, these studies either analyzed pCR as a subset of MPR or evaluated the prognostic role of nodal status in the overall population, which may have masked the prognostic heterogeneity within non-pCR MPR patients.

We also found that the number of harvested lymph nodes was an independent protective factor for prognosis in MPR patients. Each additional harvested lymph node was associated with an approximately 7% reduction in recurrence risk, and the optimal cutoff was 16 lymph nodes. Although evidence specifically evaluating the association between lymph node count and prognosis in MPR patients after NCIT remains limited, previous studies in broader NSCLC populations have demonstrated the prognostic value of lymph node evaluation. David et al. showed that harvesting fewer than 10 lymph nodes was associated with worse overall survival in patients with stage I–III NSCLC who underwent upfront surgery (22); Bahardoust et al. demonstrated a dose-response relationship between an increasing number of negative lymph nodes and improved survival (23). Liang et al. further confirmed, in patients with pN0 NSCLC who did not receive neoadjuvant therapy, that the number of examined lymph nodes was positively associated with survival and identified 16 lymph nodes as the optimal cutoff (24). This aligns closely with our findings, suggesting that this threshold may be a useful reference across different treatment settings and pathological response states. Notably, our study provides evidence for the independent prognostic value of lymph node count in the non-pCR MPR population, indicating that adequate lymph node dissection remains an important determinant of long-term recurrence risk even when the primary lesion achieves MPR.

Baseline cT3–4 stage was also identified as an independent adverse prognostic factor for RFS in MPR patients, suggesting that a greater primary tumor burden before treatment continues to affect postoperative recurrence risk after neoadjuvant chemoimmunotherapy. Although no study has specifically evaluated the prognostic value of baseline clinical T stage in MPR patients after NCIT, previous studies have shown that postoperative pathological T stage (ypT) is an independent prognostic factor in the overall population. Qu et al. reported that ypT3/4 was an independent risk factor for EFS in NSCLC patients receiving neoadjuvant chemoimmunotherapy, supporting the prognostic impact of T stage (20). Notably, because MPR is defined by ≤10% residual viable tumor in the primary lesion, ypT stage in MPR patients may become concentrated within lower pathological stages after treatment response, thereby limiting its ability to discriminate prognosis. In contrast, baseline clinical T stage retains pretreatment tumor-burden information and may better capture the effect of primary tumor burden on prognosis in MPR patients. This may reflect that baseline tumor burden influences the biology of residual disease. Specifically, patients with higher initial T stage, even after substantial downstaging, may carry a greater burden of micrometastatic clones that ultimately drive recurrence.

Based on the three independent prognostic factors (cT3–4, ypN+, and N_count ≤16), we constructed a simplified risk-stratification system that effectively identified prognostic heterogeneity within non-pCR MPR patients. Low-risk MPR patients, defined as having 0–1 risk factor, had RFS comparable to that of pCR patients (P = 0.856), whereas high-risk MPR patients, defined as having 2–3 risk factors, had significantly worse RFS than the low-risk group (P = 0.009) and outcomes similar to those of Non-MPR patients. The C-index of this stratification system was 0.637 (95% CI: 0.543–0.727), indicating modest overall discrimination, while the 24-month time-dependent AUC reached 0.702, suggesting reasonable short-term discriminatory performance. Previous studies have attempted to integrate pathological response and nodal status for prognostic stratification, but most included pCR within MPR or evaluated the entire cohort (14, 21). The present study established a risk-stratification system specifically in the non-pCR MPR population and incorporated multidimensional factors, including baseline T stage and lymph node count, providing a practical tool for prognostic assessment in this specific subgroup. From a clinical perspective, this risk-stratification system may inform individualized postoperative management strategies. Low-risk MPR patients, whose prognosis approaches that of pCR patients, may be suitable for standard surveillance strategies and may avoid overtreatment. In contrast, high-risk MPR patients achieve MPR in the primary lesion but still have a markedly increased recurrence risk due to factors such as residual nodal disease, greater baseline tumor burden, or inadequate lymph node evaluation. These patients may require closer postoperative monitoring, and the value of intensified treatment strategies should be further explored.

Regarding postoperative adjuvant therapy, our exploratory subgroup analyses did not identify a statistically significant association with RFS in any pathological response or risk-defined subgroup. This finding aligns with a recent study by Lin et al., which reported that adjuvant immunotherapy did not improve DFS or OS in a similar population of completely resected stage II–IIIB NSCLC after neoadjuvant chemoimmunotherapy (25). However, these results should be interpreted with caution. The non-randomized treatment allocation in our cohort introduces potential confounding by indication, as patients with unfavorable postoperative features may have been more likely to receive adjuvant therapy. Additionally, adjuvant regimens were heterogeneous, including immunotherapy alone or chemoimmunotherapy followed by maintenance immunotherapy, and the limited number of events within each subgroup precluded robust statistical inference. Therefore, these findings should be considered hypothesis-generating and require prospective validation, rather than being interpreted as evidence of lack of benefit.

We also analyzed recurrence patterns after NCIT. Although the overall recurrence rates differed significantly among pCR, MPR, and Non-MPR patients (15.0%, 26.1%, and 37.9%, respectively; all P < 0.05), the distribution of recurrence patterns did not significantly differ among patients who developed recurrence. The proportions of intrathoracic recurrence only, extrathoracic metastasis only, and simultaneous intrathoracic and extrathoracic recurrence were broadly similar across groups, suggesting that pathological response primarily affects the probability of recurrence rather than its anatomical pattern. Among patients with extrathoracic metastasis, the brain and bone were the most common metastatic sites: brain metastases occurred in 47.1%, 40.3%, and 41.2% of MPR, Non-MPR, and pCR patients, respectively, whereas bone metastases occurred in 35.3%, 25.8%, and 17.6%, respectively. This finding is consistent with the known metastatic characteristics of NSCLC and with previous evidence—Desai et al., in a cohort of 1,334 patients with resectable NSCLC who received neoadjuvant or adjuvant chemoimmunotherapy, similarly observed that the brain, bone, and pleura were the most common metastatic sites, and that this pattern remained consistent across neoadjuvant and adjuvant treatment cohorts (26). Notably, even among patients who achieved substantial pathological responses after NCIT, common metastatic patterns remained similar, suggesting that recurrence location is largely independent of treatment response depth. These observations may offer preliminary insights for future surveillance considerations, but given the exploratory nature of this analysis, they should be interpreted as hypothesis-generating and require further validation. Studies specifically comparing recurrence patterns across different pathological response groups after neoadjuvant chemoimmunotherapy remain limited. By using a Sankey diagram, the present study visually demonstrated the trajectory from radiological response to pathological response and recurrence pattern, providing a new perspective for understanding recurrence after NCIT.

This study has several limitations. First, because of the retrospective design, selection bias may exist. The included patients all completed neoadjuvant chemoimmunotherapy and underwent radical surgery, which may have excluded patients who failed to complete the planned treatment pathway because of severe adverse events, rapid disease progression, or unresectability at preoperative reassessment. As a result, the study population may have been enriched for patients who responded relatively well to neoadjuvant chemoimmunotherapy, and prognostic estimates may therefore be somewhat optimistic. Second, the overall follow-up duration was relatively short, with a median follow-up of 20.5 months, and some late recurrence events may not yet have occurred. Therefore, overall survival (OS) was not included in the present analysis, and longer follow-up is needed to validate the long-term prognostic value of the risk-stratification system. Third, neoadjuvant chemoimmunotherapy regimens included different PD-1/PD-L1 inhibitors and chemotherapy combinations, and postoperative adjuvant therapy was also heterogeneous, including immunotherapy alone or chemoimmunotherapy followed by maintenance immunotherapy, which may affect the precision of prognostic analyses. Moreover, adjuvant therapy was non-randomized and may have been influenced by baseline clinical characteristics and postoperative risk factors; the limited number of recurrence events within each subgroup further restricted statistical power. Therefore, these adjuvant-related findings should be interpreted as exploratory and hypothesis-generating. Additionally, the potential role of postoperative adjuvant radiotherapy could not be evaluated in this cohort, as no patient received this treatment. Its value after neoadjuvant chemoimmunotherapy, particularly in patients with multi-station N2 disease, remains to be defined in future studies. Fourth, the risk-stratification system developed in this study was only internally validated using bootstrap resampling and lacks independent external validation; therefore, its generalizability and robustness require further evaluation in other cohorts before potential clinical application. Fifth, several key biomarkers, such as PD-L1 expression and tumor differentiation, had relatively high missing rates, and emerging biomarkers such as circulating tumor DNA (ctDNA) and MRD were not included, limiting deeper understanding of prognostic heterogeneity among MPR patients. Finally, recurrence pattern analysis focused only on the first site of recurrence and did not track subsequent metastatic evolution. In addition, some microscopic distant metastases may not have been detected by imaging, potentially leading to underestimation of distant relapse risk. Additionally, imaging for brain and bone metastases was generally performed when clinically indicated rather than as part of a standardized surveillance protocol, which may have affected the observed distribution of these metastatic sites.

## 5 Conclusions

This study demonstrates that Non-pCR MPR after neoadjuvant chemoimmunotherapy is a prognostically heterogeneous state in resectable NSCLC. Stratifying these patients using baseline cT stage, postoperative nodal status, and lymph node count effectively discriminates survival outcomes, highlighting the continued necessity of thorough systematic lymph node dissection even when preoperative assessment indicates a marked treatment response. Although adjuvant therapy did not show a definitive RFS benefit in our cohort, its potential value, especially in high-risk MPR patients, warrants prospective validation and should not be interpreted as evidence of lack of benefit. Furthermore, the observed recurrence patterns across response groups, with brain and bone being frequently observed metastatic sites, provide preliminary insights into metastatic distribution after NCIT and may serve as a reference for future surveillance studies. Ultimately, this simplified risk-stratification system may provide a useful reference for prognostic assessment and individualized management. Further prospective studies are needed to validate its clinical utility in larger independent cohorts.

## Data Availability

The raw data supporting the conclusions of this article will be made available by the authors, without undue reservation.
