# Correction: Not all major pathological responses are equal: prognostic heterogeneity and risk stratification after neoadjuvant chemoimmunotherapy in resectable NSCLC

**DOI:** 10.3389/fimmu.2026.2010974

**Published:** 2026-10-09

**Authors:** Jiong Lin, Haijie Xu, Jiming Tang, Chaoquan He, Peishen Li, Xirui Lin, Jiaying Wu, Zexin Huang, Chenshu Huang, Zefeng Xie, Hansheng Wu

**Affiliations:** 1 Department of Thoracic Surgery, The First Affiliated Hospital of Shantou University Medical College, Shantou, China; 2 Shantou University Medical College, Shantou, China; 3 Department of Thoracic Surgery, Guangdong Provincial People’s Hospital (Guangdong Academy of Medical Sciences), Southern Medical University, Guangzhou, China

**Keywords:** lymph node dissection, major pathological response (MPR), neoadjuvant chemoimmunotherapy (NCIT), Non-small cell lung cancer, risk stratification

The funder “Clinical Research Special Fund of Guangdong Medical Association, grant number 2025FA-B1014”, to Hansheng Wu was erroneously omitted.

“The author(s) declared that financial support was received for this work and/or its publication. This work was supported by the Clinical Research Special Fund of Guangdong Medical Association (grant number: 2025FA-B1014) and the ‘Talent Support' Program of the First Affiliated Hospital of Shantou University Medical College. The sponsors were not involved in the study design, data collection, analysis, interpretation, manuscript writing, or the decision to submit the article for publication.”

The original version of this article has been updated.

