# Supplementary material for: Not all major pathological responses are equal: prognostic heterogeneity and risk stratification after neoadjuvant chemoimmunotherapy in resectable NSCLC
Source: Front Immunol. 2026 Sep 25;17:1925301. doi: 10.3389/fimmu.2026.1925301 (PMC13649735; doi:10.3389/fimmu.2026.1925301)
Supplement: Supplementary file 1 [file Table1.docx]

**Supplementary Files：**

**Table S1. Univariate and multivariate Cox regression for recurrence‑free survival in the entire cohort**

|  | | **Univariate** | | **Multivariate** | |
| --- | --- | --- | --- | --- | --- |
| **Variables** | **Levels** | **HR (95% CI)** | **P value** | **HR (95% CI)** | **P value** |
| **Sex** | **Male** | **Reference** | **0.474** |  |  |
|  | **Female** | **1.15 (0.79-1.69)** | **0.468** |  |  |
| **Age** | **≤65** | **Reference** | **0.332** |  |  |
|  | **>65** | **1.17 (0.85-1.61)** | **0.329** |  |  |
| **Smoking** | **Non-smoker** | **Reference** | **0.798** |  |  |
|  | **Smoker** | **0.96 (0.71-1.31)** | **0.798** |  |  |
| **BMI** | **Per 1 unit increase** | **1.04 (0.99-1.09)** | **0.166** |  |  |
| **Adjuvant therapy** | **No** | **Reference** | **0.888** |  |  |
|  | **Yes** | **0.98 (0.70-1.36)** | **0.888** |  |  |
| **Neoadjuvant cycles** | **<3** | **Reference** | **0.304** |  |  |
|  | **3-4** | **0.96 (0.66-1.39)** | **0.820** |  |  |
|  | **>4** | **1.52 (0.82-2.83)** | **0.181** |  |  |
| **Histology** | **LUAD** | **Reference** | **0.055** |  |  |
|  | **LUSC** | **0.85 (0.62-1.17)** | **0.317** |  |  |
|  | **Others** | **0.49 (0.26-0.93)** | **0.028** |  |  |
| **Differentiation** | **G1-2** | **Reference** | **0.003** |  |  |
|  | **G3** | **1.05 (0.74-1.48)** | **0.788** |  |  |
|  | **Unknown** | **0.55 (0.36-0.83)** | **0.005** |  |  |
| **Tumor site** | **Peripheral** | **Reference** | **0.161** |  |  |
|  | **central** | **0.80 (0.58-1.10)** | **0.165** |  |  |
| **cT stage** | **T1-2** | **Reference** | **0.418** |  |  |
|  | **T3-4** | **1.14 (0.83-1.54)** | **0.418** |  |  |
| **cN stage** | **N0** | **Reference** | **0.455** |  |  |
|  | **N+** | **0.86 (0.58-1.27)** | **0.447** |  |  |
| **ypT stage** | **T0** | **Reference** | **<0.001** |  |  |
|  | **T1** | **2.12 (1.43-3.16)** | **<0.001** |  |  |
|  | **T2** | **4.38 (2.71-7.05)** | **<0.001** |  |  |
|  | **T3** | **3.95 (1.95-7.98)** | **<0.001** |  |  |
|  | **T4** | **1.59 (0.49-5.17)** | **0.444** |  |  |
| **ypN stage** | **N0** | **Reference** | **<0.001** | **Reference** |  |
|  | **N+** | **2.18 (1.59-3.01)** | **<0.001** | **1.50 (1.05-2.13)** | **0.025** |
| **cTNM stage** | **I-II** | **Reference** | **0.802** |  |  |
|  | **III** | **1.05 (0.73-1.49)** | **0.803** |  |  |
| **ypTNM stage** | **0** | **Reference** | **<0.001** |  |  |
|  | **I** | **2.22 (1.44-3.45)** | **<0.001** |  |  |
|  | **II** | **2.93 (1.81-4.73)** | **<0.001** |  |  |
|  | **III** | **4.84 (2.96-7.93)** | **<0.001** |  |  |
| **RECIST** | **PD_SD** | **Reference** | **0.032** | **Reference** |  |
|  | **CR_PR** | **0.71 (0.52-0.97)** | **0.031** | **0.98 (0.71-1.37)** | **0.927** |
| **PD-L1** | **Negative** | **Reference** | **0.433** |  |  |
|  | **Strong positive** | **1.19 (0.70-2.01)** | **0.515** |  |  |
|  | **Positive** | **0.91 (0.58-1.42)** | **0.666** |  |  |
|  | **Unknown** | **0.81 (0.53-1.23)** | **0.324** |  |  |
| **Operative time** | **Per 1 unit increase** | **1.00 (1.00-1.00)** | **0.364** |  |  |
| **Blood loss** | **Per 1 unit increase** | **1.00 (1.00-1.00)** | **0.357** |  |  |
| **N_count** | **Per 1 unit increase** | **0.97 (0.95-0.99)** | **<0.001** | **0.96 (0.94-0.98)** | **<0.001** |
| **positive_N_count** | **Per 1 unit increase** | **1.11 (1.06-1.17)** | **<0.001** |  |  |
| **Response** | **pCR** | **Reference** | **<0.001** | **Reference** |  |
|  | **MPR** | **1.97 (1.18-3.29)** | **0.009** | **1.68 (0.99-2.85)** | **0.056** |
|  | **Non-MPR** | **3.19 (2.12-4.80)** | **<0.001** | **2.35 (1.47-3.76)** | **<0.001** |
| ***HR for continuous variables corresponds to per 1 unit increase (see units in Methods).** | | | | | |

**Supplementary Table S1. Univariable and multivariable Cox regression analyses for recurrence-free survival in the entire cohort.**

**Note: Hazard ratios (HRs) and 95% confidence intervals (CIs) were estimated using Cox proportional hazards regression models. Variables with P < 0.10 in univariable analysis were considered for multivariable analysis. Variables with substantial clinical overlap or potential collinearity were not simultaneously included in the final multivariable model. Specifically, ypT stage and ypTNM stage, although significantly associated with RFS in univariable analyses, were not included because of their overlap with postoperative pathological response categories and ypN status. Positive lymph node count was also not included in the multivariable model because of its close relationship with ypN status and total harvested lymph node count (N_count). The final model retained clinically interpretable variables to minimize overadjustment and avoid collinearity.**

***Table S2. Pairwise comparisons of recurrence-free survival among pathological response and risk-stratified groups***

| **Group 1** | **Group 2** | **Raw P value** | **FDR-adjusted P value** |
| --- | --- | --- | --- |
| **pCR** | **MPR_low_risk** | **0.853** | **0.856** |
| **pCR** | **MPR_high_risk** | **<0.001** | **<0.001** |
| **pCR** | **Non-MPR_ypN0** | **<0.001** | **<0.001** |
| **pCR** | **Non-MPR_ypN+** | **<0.001** | **<0.001** |
| **MPR_low_risk** | **MPR_high_risk** | **0.005** | **0.009** |
| **MPR_low_risk** | **Non-MPR_ypN0** | **0.003** | **0.007** |
| **MPR_low_risk** | **Non-MPR_ypN+** | **<0.001** | **<0.001** |
| **MPR_high_risk** | **Non-MPR_ypN0** | **0.856** | **0.856** |
| **MPR_high_risk** | **Non-MPR_ypN+** | **0.403** | **0.504** |
| **Non-MPR_ypN0** | **Non-MPR_ypN+** | **0.164** | **0.234** |

**P values were calculated using pairwise log-rank tests. FDR-adjusted P values were calculated using the Benjamini-Hochberg method. Events indicate recurrence or death.**

**Figure S1**

**Supplementary Figure S1. Prognostic performance of the MPR risk-stratification system according to histological subtype.**

Kaplan–Meier curves showing recurrence-free survival stratified by the MPR risk-stratification system in histological subgroups.
(A) Recurrence-free survival in patients with lung adenocarcinoma.
(B) Recurrence-free survival in patients with lung squamous cell carcinoma.
Low-risk MPR was defined as 0–1 risk factor, and high-risk MPR was defined as 2–3 risk factors based on baseline cT3–4 stage, postoperative nodal positivity, and N_count ≤16. Log-rank P values are shown in each panel.

**Figure S2**

**Supplementary Figure S2. Center-specific Kaplan–Meier analyses of recurrence-free survival according to the proposed risk stratification system.**
(A) Kaplan–Meier analysis in the larger cohort (n=512).
(B) Kaplan–Meier analysis in the smaller cohort (n=76).
Patients were categorized into four groups: pCR, MPR low-risk, MPR high-risk, and Non-MPR. In the larger cohort, the prognostic pattern was consistent with the overall population. In the smaller cohort, the same directional trend was observed, although differences between groups did not reach statistical significance, likely due to the limited sample size and event numbers. Pairwise comparisons versus pCR were performed using FDR-adjusted log-rank tests.

**Figure S3**

**Supplementary Figure S3. Exploratory Kaplan–Meier analysis of recurrence-free survival according to postoperative adjuvant therapy type.**
(A) RFS comparison between patients receiving adjuvant immunotherapy alone and those without adjuvant therapy (log-rank P = 0.161).
(B) RFS comparison between patients receiving adjuvant chemoimmunotherapy followed by maintenance immunotherapy and those without adjuvant therapy (log-rank P = 0.162).

These analyses are exploratory and were performed to evaluate the association between different postoperative adjuvant therapy types and RFS. Given the limited sample sizes and non-randomized treatment allocation, these findings should be interpreted cautiously.
